# Supplementary material for: TTYH1 and TTYH2 Serve as LRRC8A-Independent Volume-Regulated Anion Channels in Cancer Cells
Source: Cells. 2019 Jun 9;8(6):562. doi: 10.3390/cells8060562 (PMC6628158; doi:10.3390/cells8060562)
Supplement: Supplementary file 1 [file cells-08-00562-s001.zip › Supplementary Figures.pdf]

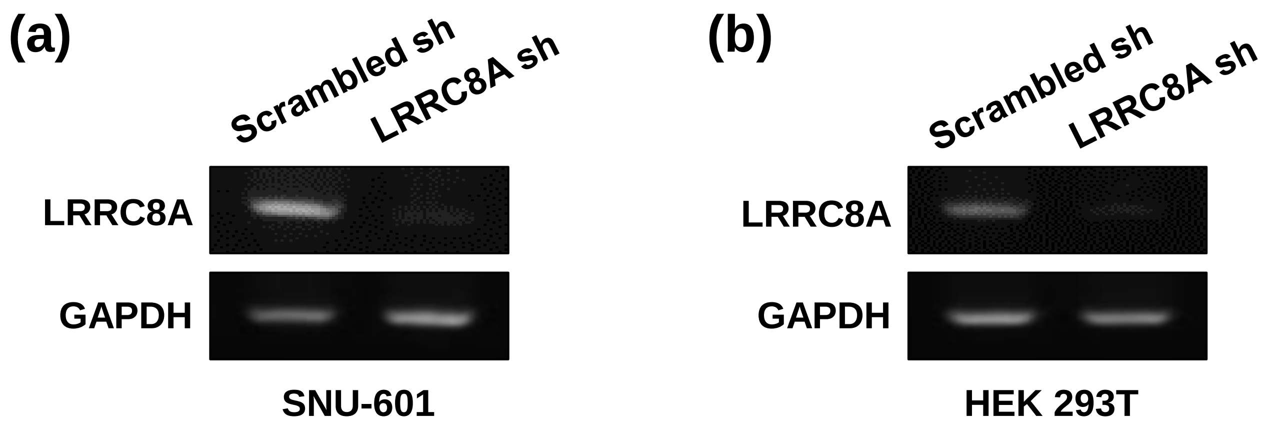

Supplemental Figure 1. Validations of *LRRC8A* shRNA. Validation of LRRC8A shRNA by RT-PCR. Scramble or *LRRC8A* shRNA was transfected into (a) SNU-601 and (b) HEK293T cells. *GAPDH* was used as a loading control.

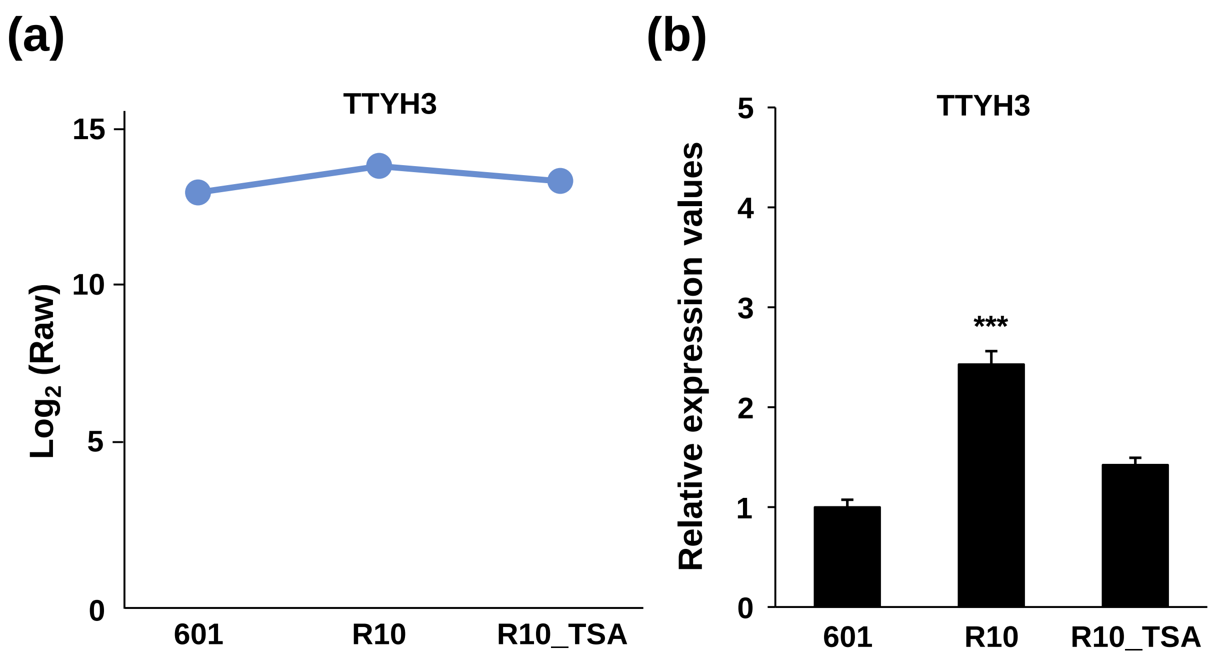

Supplemental Figure 2. TTYH3 is expressed in a different pattern from TTYH1 and TTYH2. (a) Dot plots represent the expression levels of TTYH3 in SNU-601, R10, and R10+TSA cells, calculated from array data. (b) Relative expression levels of *TTYH3* mRNA in SNU-601, R10, and TSA-treated R10 cells. Data are expressed as means ± SEM (*** $P < 0.001$).
