# Supplementary material for: TTYH1 and TTYH2 Serve as LRRC8A-Independent Volume-Regulated Anion Channels in Cancer Cells
Source: Cells. 2019 Jun 9;8(6):562. doi: 10.3390/cells8060562 (PMC6628158; doi:10.3390/cells8060562)
Supplement: Supplementary file 1 [file cells-08-00562-s001.zip › Supplementary Table 1.pdf]

Supplementary Table 1. Fold changes of the chloride channel genes from our microarray data.

	Filter: 118		Fold change		Raw (log2)		
	Systematic	Gene Symbol	R10 con/ 601	R10 TSA/ 601	601	R10 con	R10 TSA
1	A_23_P101683	CLC	0.336	15.259	5.778	3.790	9.699
2	A_23_P104996	BEST1	2.430	1.101	6.192	7.058	6.320
3	A_23_P109895	SLC26A6	1.145	1.559	9.719	9.500	10.350
4	A_23_P112801	CHP	0.792	0.634	15.300	14.549	14.632
5	A_23_P116642	SLC26A10	1.345	0.867	8.490	8.503	8.274
6	A_23_P117873	CHRM5	2.814	2.536	6.490	7.568	7.823
7	A_23_P122134	NMUR2	2.577	4.964	5.669	6.620	7.970
8	A_23_P123228	SLC26A3	1.496	1.517	6.450	6.617	7.041
9	A_23_P124476	CLCN3	0.625	1.298	11.121	10.028	11.488
10	A_23_P125078	SLC26A11	0.868	0.896	8.606	7.988	8.438
11	A_23_P125435	GABRB1	1.177	1.338	3.946	3.767	4.356
12	A_23_P125475	GABRQ	5.245	1.416	3.834	5.811	4.326
13	A_23_P125618	GABRA3	2.372	3.648	5.345	6.176	7.202
14	A_23_P127995	CLNS1A	0.886	0.675	13.912	13.322	13.334
15	A_23_P129334	CLCN7	1.738	1.439	6.583	6.966	7.098
16	A_23_P132845	CLCN2	0.237	16.797	7.556	5.062	11.616
17	A_23_P135499	CLIC4	0.938	0.552	10.864	10.357	9.997
18	A_23_P156261	GLRA1	2.184	1.276	4.384	5.096	4.725
19	A_23_P159775	GABRE	0.704	0.913	12.741	11.820	12.600
20	A_23_P16225	BEST2	0.916	1.275	5.701	5.160	6.041
21	A_23_P167121	GABRA2	1.628	1.519	3.061	3.349	3.653

22	A_23_P19852	IQCE	0.448	0.216	11.442	9.869	9.221
23	A_23_P201248	SLC26A9	0.580	0.608	5.071	3.870	4.343
24	A_23_P215720	CFTR	0.182	0.031	9.479	6.610	4.452
25	A_23_P23029	BSND	1.364	0.942	8.971	9.004	8.875
26	A_23_P256754	GABRR1	0.661	0.157	6.105	5.094	3.424
27	A_23_P259189	CLIC4	0.305	0.590	11.401	9.272	10.630
28	A_23_P30884	CLIC1	0.636	0.272	13.100	12.033	11.212
29	A_23_P30950	SLC26A8	1.134	1.788	3.303	3.069	4.131
30	A_23_P309720	GABRD	2.157	2.674	6.448	7.143	7.857
31	A_23_P311895	CLIC5	0.608	0.205	5.730	4.597	3.434
32	A_23_P31903	VPS28	0.214	0.342	15.294	12.656	13.736
33	A_23_P323801	BEST3	1.113	1.228	3.603	3.343	3.889
34	A_23_P328545	GABRP	0.527	0.876	8.674	7.335	8.474
35	A_23_P331560	SLC26A4	1.256	1.306	3.311	3.225	3.686
36	A_23_P331895	TTYH3	2.161	1.240	12.598	13.295	12.898
37	A_23_P335495	ANO7	2.070	1.689	11.964	12.599	12.710
38	A_23_P340868	GLRA3	1.335	1.405	3.731	3.733	4.211
39	A_23_P34382	CLCA3P	2.255	1.400	5.194	5.953	5.670
40	A_23_P35725	ANO3	5.337	2.019	11.214	13.215	12.217
41	A_23_P360924	CLDN17	0.704	0.700	4.054	3.134	3.529
42	A_23_P385067	CLIC6	1.307	1.336	3.236	3.207	3.643
43	A_23_P389118	ANO6	1.825	0.647	12.648	13.101	12.010
44	A_23_P397248	CLCA2	1.559	1.853	5.980	6.206	6.859
45	A_23_P409093	ANO4	0.972	1.147	4.084	3.628	4.271
46	A_23_P416774	CLIC5	0.535	0.108	7.461	6.143	4.245

47	A_23_P41847	GABRA6	1.417	1.424	3.501	3.589	4.001
48	A_23_P41908	FAM114A2	1.656	3.371	5.219	5.532	6.962
49	A_23_P436618	GABRA5	1.504	1.102	3.283	3.457	3.413
50	A_23_P45751	CLCA4	1.174	1.854	6.145	5.962	7.025
51	A_23_P50815	TTYH1	0.101	2.590	10.971	7.251	12.333
52	A_23_P51217	CLCA1	1.853	2.176	6.139	6.615	7.251
53	A_23_P58763	PELO	0.962	1.066	10.920	10.449	11.002
54	A_23_P59772	CLCN1	0.727	0.978	7.220	6.345	7.178
55	A_23_P66432	TTYH2	0.092	0.501	6.942	3.090	5.936
56	A_23_P69020	GABRR3	0.454	0.457	7.659	6.105	6.519
57	A_23_P73413	SLC26A7	1.078	1.137	3.235	2.929	3.410
58	A_23_P78750	SLC17A7	1.040	10.761	7.271	6.913	10.688
59	A_23_P80954	SLC26A1	1.386	1.293	7.596	7.652	7.956
60	A_23_P93105	GABRR2	1.033	1.654	4.863	4.496	5.579
61	A_24_P100996	ANO5	1.130	1.172	3.304	3.066	3.524
62	A_24_P16913	ABCC4	0.292	0.181	5.971	3.780	3.494
63	A_24_P191312	SLC1A4	0.620	0.762	8.623	7.519	8.221
64	A_24_P222599	PDPK1	2.719	2.499	9.356	10.384	10.667
65	A_24_P224966	CLCNKB	0.516	0.794	6.727	5.356	6.384
66	A_24_P225010	RPS15	1.352	1.009	15.349	15.370	15.351
67	A_24_P2361	CLCNKA	1.128	1.171	7.440	7.198	7.657
68	A_24_P266285	IQCE	0.905	1.419	9.291	8.733	9.786
69	A_24_P29117	SLC26A1	0.799	0.420	6.586	5.848	5.326
70	A_24_P302374	CLCN6	0.954	0.549	8.973	8.490	8.099
71	A_24_P367410	GABRQ	2.268	2.305	5.291	6.058	6.486

72	A_24_P381962	AP1G1	1.336	1.145	11.660	11.663	11.844
73	A_24_P404868	GLRA4	1.081	0.448	6.048	5.746	4.880
74	A_24_P8109	ANO9	0.498	0.475	10.418	8.997	9.335
75	A_24_P830690	PDPK1	3.769	1.218	6.962	8.461	7.236
76	A_24_P87036	ANO1	0.802	0.056	13.042	12.309	8.886
77	A_24_P87931	APOL1	0.585	0.200	16.115	14.927	13.782
78	A_32_P141238	ANO2	4.663	5.449	4.037	5.843	6.472
79	A_32_P204137	GABRA4	0.997	1.043	3.345	2.926	3.395
80	A_32_P25514	GABRG2	7.424	110.858	3.207	5.685	9.990
81	A_32_P381593	SLC26A7	2.572	2.055	5.743	6.691	6.772
82	A_32_P73452	ANO8	1.151	0.522	6.059	5.847	5.110
83	A_32_P89899	GABRG1	1.350	1.422	3.747	3.765	4.244
84	A_33_P3210805	GABRG3	7.529	3.448	4.212	6.709	5.987
85	A_33_P3224250	CLCC1	1.231	1.423	6.991	6.877	7.490
86	A_33_P3228435	FXYD1	2.051	1.845	6.873	7.495	7.746
87	A_33_P3228450	FXYD3	0.291	0.092	12.968	10.774	9.508
88	A_33_P3228455	FXYD3	0.753	0.848	8.922	8.098	8.674
89	A_33_P3228460	FXYD3	0.653	0.233	15.819	14.789	13.710
90	A_33_P3228466	FXYD3	0.166	0.086	11.560	8.557	8.012
91	A_33_P3244808	BEST4	2.196	1.776	11.547	12.267	12.365
92	A_33_P3247342	ANO7	0.235	0.532	11.099	8.597	10.177
93	A_33_P3249888	BEST3	0.899	0.576	4.341	3.773	3.534
94	A_33_P3277075	GABRB3	1.300	13.446	3.910	3.874	7.649
95	A_33_P3277447	SLC26A2	2.111	4.060	12.595	13.258	14.606
96	A_33_P3285545	CLDN4	0.285	0.181	13.687	11.463	11.210

97	A_33_P3286804	ANO6	1.043	1.058	3.399	3.045	3.470
98	A_33_P3287108	TMEM132E	0.999	5.842	3.515	3.100	6.052
99	A_33_P3328426	ANO10	0.824	1.331	8.648	7.954	9.050
100	A_33_P3332052	SLC26A11	1.449	0.526	6.472	6.592	5.534
101	A_33_P3336686	CLIC3	0.297	0.896	10.580	8.416	10.411
102	A_33_P3336715	GABRB2	1.720	3.414	4.131	4.499	5.893
103	A_33_P3337049	GLRA2	1.362	1.383	3.408	3.440	3.866
104	A_33_P3367596	CLCN4	2.224	5.141	4.613	5.352	6.965
105	A_33_P3371224	ANO10	1.169	1.147	6.973	6.784	7.160
106	A_33_P3373364	CLIC4	0.366	1.117	9.289	7.425	9.439
107	A_33_P3373388	GABRA1	1.043	1.218	3.308	2.953	3.582
108	A_33_P3387943	GLRB	1.675	1.682	3.437	3.766	4.178
109	A_33_P3392177	CLIC5	0.816	0.500	13.148	12.439	12.138
110	A_33_P3400699	SLC26A5	6.829	5.314	6.399	8.756	8.799
111	A_33_P3400700	SLC26A5	1.239	3.949	3.251	3.145	5.222
112	A_33_P3412149	CLCC1	1.096	0.852	16.085	15.803	15.844
113	A_33_P3412160	CLCC1	3.687	0.409	8.153	9.621	6.855
114	A_33_P3415087	CLCN5	0.942	1.141	7.661	7.160	7.841
115	A_33_P3415092	CLCN5	0.688	0.401	11.424	10.469	10.095
116	A_33_P3415097	CLCN4	0.350	4.042	9.281	7.350	11.286
117	A_33_P3415113	CLCN6	0.766	0.114	6.905	6.106	3.761
118	A_33_P3775848	CLIC2	0.309	0.707	5.655	3.546	5.145
